# Editorial Notes and Miscellany

**Published:** 1912-06

**Authors:** 


### Editorial Notes and Miscellany.

Tuberculosis Sanitarium No. 1 at Carlsbad, Texas, has been completed and will be formally opened June 27th. A barbecue and speeches will be the program. Hon. T. H. McGregor, who fathered the bill creating two sanatoria, will deliver the dedicatory address. The total appropriation for the two establishments and equipment was $140,000. The Commissioners are Hon. F. C. Hume, Frank Bushick and Mr. Connally. The medical officers to have charge have not yet been appointed.

Murder and the Saloon.—Within four months four shocking murders have been committed in fair Austin, the fair Capital City, three of which were the result of whisky and the saloon influences. But—don't abolish the saloon; it will "injure business." Better children starve, hearts break, chains clank and maniacs rave than to "injure business." What a commentary on our "civilization!"

